# Gestational choriocarcinoma diagnosed with spontaneous splenic rupture after pregnancy induced by in vitro fertilization: a case report

**DOI:** 10.1186/1757-1626-2-7518

**Published:** 2009-05-15

**Authors:** Mehmet Yürüyen, Ozcan Yildiz, Cigdem Papila, Nukhet Tuzuner

**Affiliations:** 1 Department of Medical Oncology, Istanbul UniversityCerrahpasa Medical Faculty, KocamustafapasaTurkey; 2 Department of Pathologym, Istanbul UniversityCerrahpasa Medical Faculty, KocamustafapasaTurkey

## Abstract

Gestational choriocarcinoma is a highly malignant tumor of trophoblastic cells with a propensity to metastasize to various sites including lungs, vagina, brain, liver, kidney, and gastrointestinal tract, in descending order of frequency. A 29-year-old Caucasian woman presented to the hospital as an emergency with abdominal pain, hypotension, nausea and vomiting at about 6th month of a twin delivery after in vitro fertilization. Our case probably represents the first case of metastatic choriocarcinoma developed after in vitro fertilization, whether associted with or not, which was cured successfully after chemotherapy.

## Introduction

Gestational choriocarcinoma is a highly malignant tumor of trophoblastic cells with a propensity to metastasize to various sites including lungs, vagina, brain, liver, kidney, and gastrointestinal tract, in descending order of frequency. The ultimate cause of gestational trophoblastic disease is claimed to be genetic in origin. No environmental etiological factor has been implicated up to now apart from deficient vitamin A precursor carotene in diet [1]. The first case of choriocarcinoma after in vitro fertilization (IVF) was reported by Flam F et al. [2] and the second by Scott P et al [3].

We hereby report the third case diagnosed with spontaneous splenic rupture, a peculiar mode of presentation. Being metastatic at presentation, it is also the first case ever reported in the English literature. We also discuss possible etiopathogenic role of IVF with gestational trophoblastic disease.

## Case presentation

A 29-year-old Caucasian woman presented to the hospital as an emergency with abdominal pain, hypotension, nausea and vomiting at about 6th month of a twin delivery.

Sixteen months previously she had been operated on due to vaginal bleeding. Pathologic examination at that time revealed necrotic chorion villi and chronic endometritis. Thirty-nine months previously she had been started on an in vito fertilization protocol due to the diagnosis of infertility. The regimen consisted of recombinat FSH (Puregon, Organon, Turkey), human menopausal gonadotropin (Humagon, Organon, Turkey), human chorionic gonadotropin (Pregnyl, Organon, Turkey), leuprolide acetate (Lucrin, Abbott Laboratories, Turkey) and LHRH analog (Suprecur, Aventis, Turkey). She was continued on this treatment for two months and stopped when doctors told her husband had azospermia. One month later another 2 months hormonotherapy failed to generate oocytes. Seven months later she was again started on gestoden and ethynyestradiol (Ginera, Schering, Turkey) together with leuprolide acetate and recombinant FSH. After successful collection of oocytes in vitro fertilization was achieved and she became pregnant with twins. Pregnancy was supported with progestogen tablets up until 10th week of gestation. She had nausea and vomiting starting from the 4th week till 24th week of gestation. She delivered two healthy babies at term. Postpartum bleeding continued for two months and dilatation & curettage were performed due to placental retention and no abnormal pathology was noted at that time. Irregular menses occured thereafter. No abnormality was detected apart from a left sided ovarian cyst. Three months before presentation symptoms of left sided abdominal pain, nausea and vomiting started. Physical examination and laboratory tests were reported to be within normal limits. Proton pump inhibitors and antacids gave no relief.

When she presented to the emergency room of the local hospital, rigidity and rebound tenderness were detected on abdominal examination. Abdominal ultrasonography revealed intraabdominal fluid and solid mass in the spleen. Splenectomy was performed with the diagnosis of spontaneous splenic rupture and she recovered uneventfully. The diagnosis of a local pathologist was diffuse large cell non-Hodgkin lymphoma (NHL). She was referred to the oncology clinic of our University Hospital.

Paraffin blocks were obtained and transferred to our hematopathology department. Microscopic features were inconsistent with NHL. However, there was a necrotic and hemorrhagic tumor composed of pleomorphic cytokeratin positive epithelial cells with round nuclei and large cytoplasm. These cells were surrounded with cytokeratin(-) and β-HCG(+) sincyciotrophoblastic cells. The definitive pathologic diagnosis was metastatic choriocarcinoma.

Computerized tomography (CT) scan of the abdomen and pelvis showed bilateral ovarian cysts, a 2 cm left sided renal lesion, and bilateral inguinal lymph nodes. CT scan of the chest revealed aortopulmonary lymph nodes less than 1 cm in diameter and minor parancymal nodules less than 5 mm in diameter. Cranial magnetic resonance imaging was normal. Plasma beta-human chorionic gonadotropin (β-HCG) level was found be 7991 mIU/mL (normal value: 0-3 in males and nonpregnant females). She was treated with 4 cycles of BEP regimen (cisplatin, bleomycin and etoposide) starting from 8 months after antecedent pregnancy. Her β-HCG level dropped to 18,3 mIU/mL after the first cycle and <1 mIU/mL after the second cycle and remained low thereafter. She has no sign of recurrence for the last 25 months of follow-up.

## Discussion

Gestational choriocarcinoma is the most malignant form of a group of tumors including complete and partial molar pregnancy, invasive mole, placental site trophoblastic tumor, and choriocarcinoma. They are collectively known as gestational trophoblastic disease. Although choriocarcinoma has a very high propensity to metastasize to various sites including brain it has also a very high cure rate. Nongestational choriocarcinoma also occurs and is usually resistant to therapy [4]. The precise molecular pathogenesis of gestational trophoblastic disease is yet to be elucidated. Genetics has a well established role. Kajii reported that all chromosomes in true moles (complete moles) are of paternal origin (androgenetic) [5]. Cytogenetic analysis revealed that in 90% of the time trophoblasts have 46XX diploid pattern all derived from the sperm. In complete moles no maternal DNA is present in the ovum. Sometimes two sperms can fertilize one empty ovum. In both cases embryonic development does not occur. Choriocarcinomas arise much more frequently from complete moles (2%).

Ethnic origin, age, diet and oral contraceptives have been implicated as a risk factor for gestational trophoblastic disease (GTD). On the other hand, there are several case reports showing development of GTD after IVF. An attempt to explain the relationship between infertility, its treatment and GTD by Bates et al. did not find a potential association [6].

Franceschi et al. reported that women who used fertility drugs were not at increased risk of developing ovarian cancers compared with women who did not [7]. One year later Rossing found that only clomiphene citrate may be associated with ovarian cancer but only after using it more than 12 months [8]. A study based on a large cohort of IVF patients reported incresed incidence of uterine sarcoma but not choriocarcinoma [9].

In conclusion, our case probably represents the first case of metastatic choriocarcinoma developed after in vitro fertilization, whether associted with or not, which was cured successfully after chemotherapy.

## List of abbreviations

CT: Computerized tomography
GTD: gestational trophoblastic disease
FSH: Follicle stimulating hormone
IVF: in vitro fertilization
NHL: non-Hodgkin lymphoma
β-HCG: beta-human chorionic gonadotropin
